# In Vivo Toxicity of Solasonine and Its Effects on cyp450 Family Gene Expression in the Livers of Male Mice from Four Strains

**DOI:** 10.3390/toxins10120487

**Published:** 2018-11-23

**Authors:** Youbao Zhong, Shanshan Li, Liling Chen, Zhiyong Liu, Xiaoquan Luo, Peng Xu, Lai Chen

**Affiliations:** 1 Laboratory Animal Research Center for Science and Technology, Jiangxi University of Traditional Chinese Medicine, 1688 Meiling Road, Nanchang 330004, China; zhong-youbao@foxmail.com (Y.Z.); shines-li@yahoo.com (S.L.); lilingchen309@foxmail.com (L.C.); liuzhiyong0791@163.com (Z.L.); lxqdjl@126.com (X.L.); xp0420@163.com (P.X.); 2 Key Laboratory of Pharmacology of Traditional Chinese Medicine in Jiangxi, Nanchang 330004, China

**Keywords:** solasonine, genetic background, in vivo toxicity, cyp450 family gene expression

## Abstract

Solasonine was reported to inhibit tumour cell growth in several different models. The in vivo toxicity of solasonine, the effects of genetic background on its toxicity, and its possible roles in regulating the expression of cyp450 family genes were still unclear and required characterisation. Here, Horn’s assays were performed on male mice from four different strains, and the expression of cyp450 family genes in their livers was examined by RT-PCR and ELISA. Mice treated by intraperitoneal injection with high levels of solasonine showed immediate post-excitatory depression, intraperitoneal tissue adhesion, and dissolving of cells in the liver. Furthermore, these four mouse strains showed different toxicological sensitivity to solasonine. The strains, in decreasing order of LD50 value, rescuing speed of body weight, and more severe pathological symptoms, were KM, ICR, C57BL/6, and BALB/c. Interestingly, more cyp450 genes were downregulated at the mRNA and/or protein level in the livers of male mice from C57BL/6 or BALB/c strains than those from KM or ICR strains. These results suggest that (1) Solasonine has hepatic toxicity and downregulates cyp450 genes expression at transcriptional and/or post-transcriptional levels; (2) Genetic background is an important factor which can affect the in vivo toxicity; (3) Downregulation of cyp450 gene expression in the liver may be a clue to help understand whether or not a given strain is sensitive to solasonine; (4) Influences on the expression of cyp450 genes should be considered when using solasonine alone, or in combination with other drugs.

## 1. Introduction

Genetic background can influence clinical symptoms. Mice heterozygous for MDM2 (mouse double minute 2 homolog) and MDM4 (mouse double minute 4 homolog) all die during embryogenesis when bred on the 129-mouse background, while the majority of mice with the same genotype do not show obvious developmental defects, and could mature to adulthood on the C57BL/6 background [1]. Genetic background can also influence the pharmacological and/or toxicological effects of drugs used in clinical treatments, as the same drugs at the same doses may cause different pharmacological function/toxicity. Alternatively, in order to obtain the same or similar function/toxicity, drug doses should be adjusted under different genetic backgrounds [2,3,4,5]. Inconsistent expression of the same genes due to different genetic background may be a reasonable explanation for the genetic background-dependent phenotypes [6], suggesting that genetic background should be considered for clinical drug usage.

Drug biotransformation regulated by cyp450 (cytochrome P450 proteins) family genes [6,7,8,9], is important for the pharmacological and toxicological effects of drugs [10]. Interestingly, the expression and activity of cyp450 family genes can be regulated by many drugs which, in turn, can affect the biotransformation and pharmacological or toxicological functions of the drugs themselves [9,11,12,13,14]. Currently, more than 57 human and 103 mouse cyp450 family genes have been identified from genome analysis [15,16], and more than 78 cyp450 genes have been characterised in mice [17]. Members of the cyp450 gene family are usually expressed at various levels. For studies on drug biotransformation and function, cyp1a2, cyp2e1, and cyp3a11, which are expressed at high levels in human and murine livers, are often employed [9,17,18,19]. Of note, basal levels of the expression of genes in the cyp450 family were reported to be dependent on factors such as gender [17,18], diseases [20], stresses [21], or age [22]. Here, we hypothesise that genetic background is a non-ignorable factor that can modify the pharmacological and toxicological function of drugs by modulating the induced expression of cyp450 genes upon drug administration.

Solasonine is a key component of the anticancer effects of *Solanum nigrum* Linn., which grows wild around the world, and is one of the ten major anticancer drugs used in traditional Chinese medicine [23,24]. Solasonine was reported to inhibit the growth of many different tumour cells, including human glioma cells (U87 [25], U251 [25,26], U118 [25], MO59J [26], U343 [26]), human colon carcinoma cells (HT29 [26]), human cervical adenocarcinoma cells (Hela [26]), human hepatocellular liver carcinoma cells (HepG2 [26]), human breast cancer cells (MCF-7 [27,23], MDA-MB-231 [27,23]), human lung cancer cell lines (H446 [28], A549 [23]), human gastric cancer cells (MGC803 [23]), human cholangiocarcinoma epithelial cells (QBC939 [29]), murine melanoma cells (B16F10 [26], B16 [30]) and murine Lewis lung cells [24], probably by activating BAX [28,29] or inhibiting the activity of the Hh pathway [31]. In a recent study, we found that high levels of solasonine exhibited in vivo toxicity in FVB mice [32]. However, it is unknown whether the toxicity of solasonine in these mice is FVB background specific, or whether this observation is a general phenomenon in mice with different genetic backgrounds. Moreover, as important modulators of the pharmacological and toxicological function of drugs, it is also unclear whether expression of cyp450 family genes could be regulated by solasonine. Here, we compared the in vivo toxicity of solasonine in male mice from four different strains—KM, ICR, C57BL/6, and BALB/c—by Horn’s assays [33], and the effects of solasonine on regulating the expression of genes in the cyp450 family in the livers of male mice from these four different strains were also evaluated.

## 2. Results

### 2.1. LD50 Values of Solasonine in Four Different Mouse Strains in the First Week after Treatment

**Figure 1:**
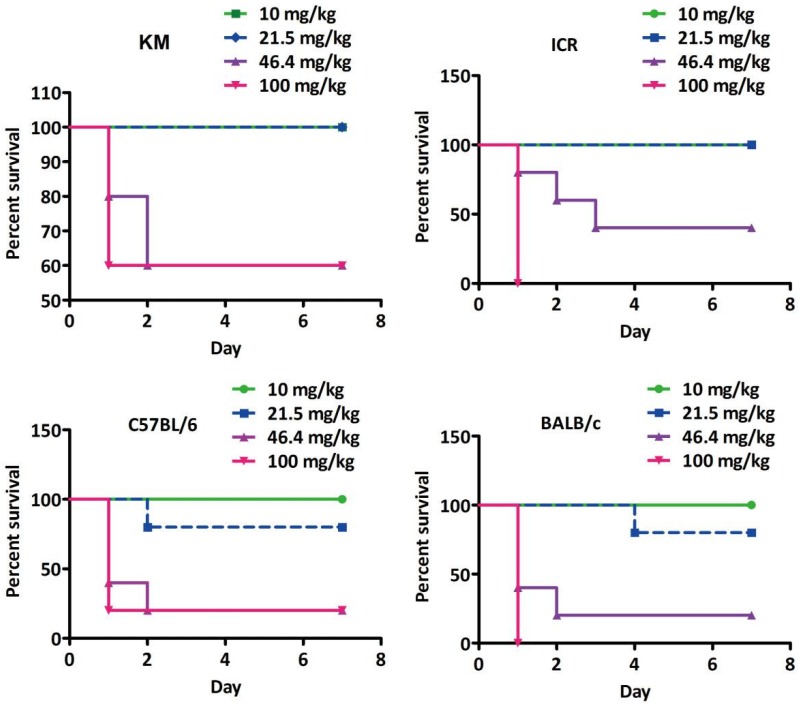
Survival curves of male mice from four strains treated by intraperitoneal injection with solasonine. Five mice in each group of KM, ICR, C57BL/6, or BALB/c strains were treated by intraperitoneal injection with solasonine at dose of 10, 21.5, 46.4, or 100 mg/kg. Then, the number of deceased mice was counted each week.

To characterise the in vivo toxicity of solasonine and to reveal the influence of genetic background on its toxicity, mice from four different strains, KM, ICR, C57BL/6, and BALB/c, were evaluated with Horn’s assays [33]. In order to exclude the influences of sex as shown in References [17,18], only male mice were used in this study. In addition, thanks to the extremely low absolute bioavailability of oral administration [34], mice were administered with solasonine by intraperitoneal injection. According to Reference [25] and Horn’s assay (*n* = 5, 2.15×) [33], the in vivo toxicity of solasonine was evaluated at the doses 10, 21.5, 46.4, and 100 mg/kg. Except for those in the 10 mg/kg groups and vehicle groups, all treated mice showed post-excitatory depression in 1.5 min (Supplemental Movie S1). In the first week after solasonine treatment, the number of mice that died at the doses of 100, 46.4, 21.5, and 10 mg/kg, respectively, were 2, 2, 0, 0 for KM; 5, 3, 0, 0 for ICR; 4, 4, 1, 0 for C57BL/6; and 5, 4, 1, 0 for BALB/c mice (Table 1 and Figure 1). According to the table of Horn’s assay (*n* = 5, 2.15×) [33], LD50 values of solasonine in KM, ICR, C57BL/6, and BALB/c strains were >43, 43, 34.8, and 31.6 mg/kg, respectively (Table 1). This suggests that the order of sensitivity to solasonine from weak to strong was KM, ICR, C57BL/6, and then BALB/c.

### 2.2. Body Weight of Mice from Four Different Strains in the First Week after Intraperitoneal Injection of Solasonine

**Figure 2:**
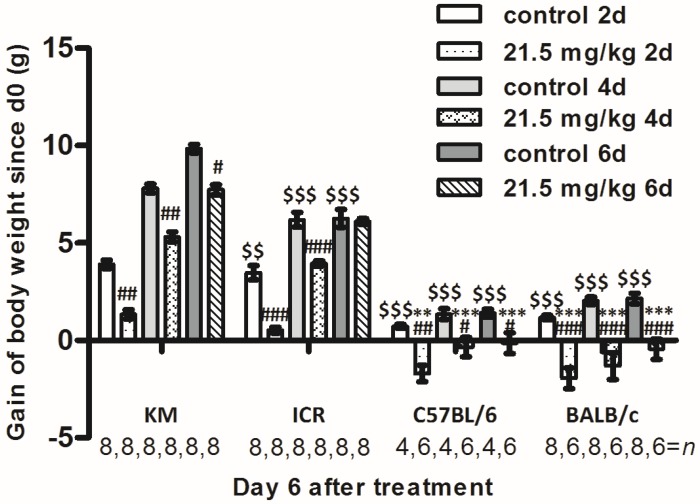
Changes in the body weights of male mice from four strains after intraperitoneal injection with solasonine or vehicle (control). Mice of KM, ICR (1/9 died within 6 days after solasonine administration), C57BL/6, and BALB/c (3/9 died within 6 days after solasonine administration) strains were treated by intraperitoneal injection with solasonine at a dose of 21.5 mg/kg. Body weights were weighed on Day 0 (the day mice were treated, the same below), Day 2, Day 4, and Day 6 after treatment. #: compared to vehicle group from the same strain on the same day, #: *p* < 0.05, ##: *p* < 0.01, ###: *p* < 0.001; $: compared to vehicle group of KM strain on the same day, $$: *p* < 0.01, $$$: *p* < 0.001; *: compared to 21.5 mg/kg group of the KM strain on the same day, **: *p* < 0.01,***: *p* < 0.001.

As 21.5 mg/kg was the lowest dose of solasonine that showed post-excitatory depression in mice from different strains and caused moderate animal death compared to higher doses (Table 1 and Figure 1), it was chosen for the following experiments. After injecting 21.5 mg/kg of solasonine or vehicle, the body weights of the treated mice were measured immediately, 2 days, 4 days, and 6 days later. It was found that the body weights of solasonine treated mice were lower, but increased continuously, compared to the control mice in each strain (Figure 2, Figure S1). The body weights of solasonine-treated KM mice became very close to the control KM mice 6 days after solasonine injection (Figure 2, Figure S1). A similar phenomenon was observed in the ICR mice (Figure 2, Figure S1). In C57BL/6 or BALB/c strains, the mouse body weight decreased remarkably, and was always lower compared to control mice after solasonine injection (Figure 2, Figure S1). Of note, compared to control mice, mice from the C57BL/6 strain showed faster gains in body weight within the 6 days after solasonine injection than those of BALB/c mice (Figure 2, Figure S1). This suggests that the speed of rescue of body weight, from fastest to slowest, was KM or ICR, C57BL/6, then BALB/c.

### 2.3. Ratios of Liver and Spleen Weight to Body Weight in Mice of Four Different Strains Six Days after Solasonine Injection

**Figure 3:**
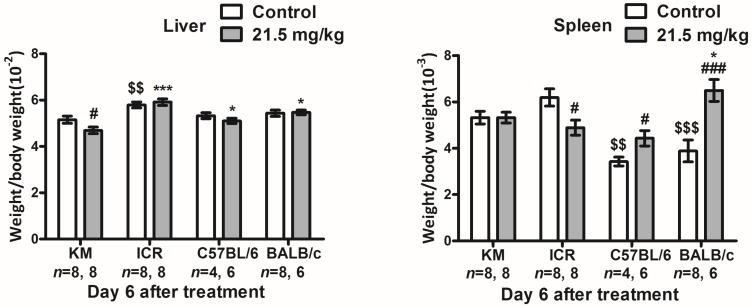
Changes in the ratios of liver and spleen weight/body weight of male mice from four strains six days after intraperitoneal injection with solasonine or vehicle (control). After body weight was evaluated, liver and spleen were dissected from mice of KM, ICR, C57BL/6, and BALB/c strains, dried by absorbent paper, and weighed six days after treatment with solasonine at a dose of 21.5 mg/kg or vehicle. Then, the ratio of spleen or liver weight/body weight was counted. #: compared to vehicle group from the same strain on the same day, #: *p* < 0.05, ###: *p* < 0.001; $: compared to vehicle group of KM strain on the same day, $$: *p* < 0.01, $$$: *p* < 0.001; *: compared to 21.5 mg/kg group of the KM strain on the same day, *: *p* < 0.05.

Six days after intraperitoneal injection of 21.5 mg/kg of solasonine, the ratios of spleen weight to body weight and liver weight to body weight were examined in all four strains. Compared to the vehicle group, no significant difference was observed in the ratio of spleen weight/body weight in KM mice (Figure 3, Spleen). However, the spleen weight/body weight ratio was decreased in ICR mice, while it was increased in C57BL/6 or BALB/c mice compared to control (Figure 3, Spleen). The ratio of liver weight/body weight was slightly decreased in KM mice (Figure 3, Liver). Interestingly, no significant difference in liver weight/body weight ratios was observed in ICR, BALB/c, or C57BL/6 mice (Figure 3, Liver). This suggests that compared to control, and related to its body weight, the spleen weight of the solasonine group grew at slower rate in the ICR strain, and at a faster rate for C57BL/6 and BALB/c. Liver weight grew at a slightly lower rate for the KM strain.

### 2.4. Pathological Changes in Mice Treated with Solasonine

**Figure 4:**
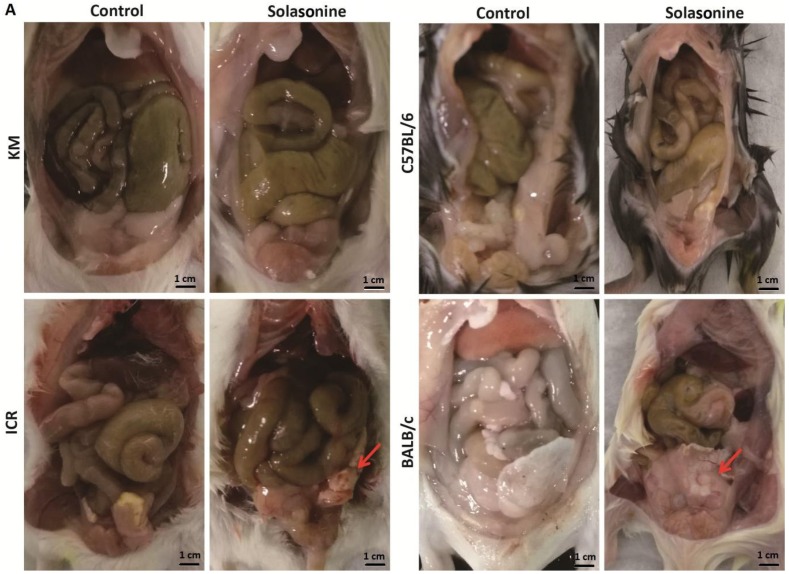

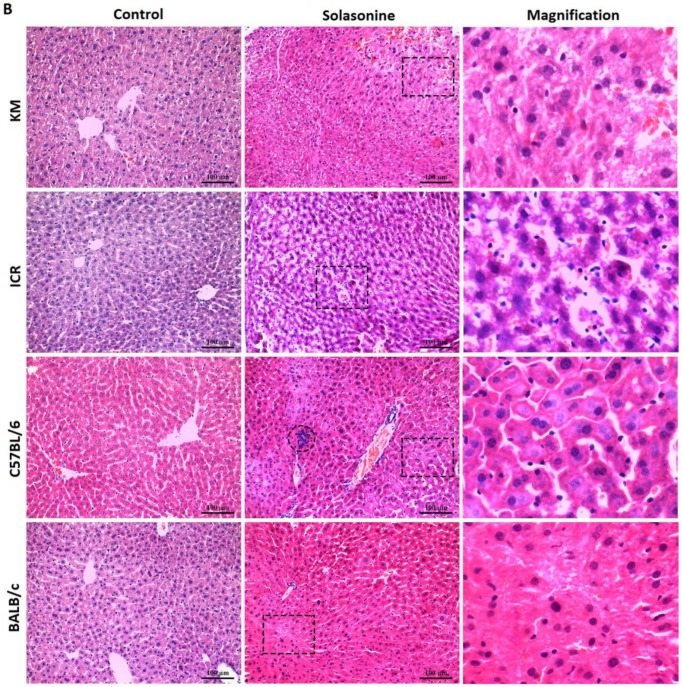
Pathological changes in mice six days after intraperitoneal injection with solasonine at a dose of 21.5 mg/kg or a vehicle (control). (**A**) Intraperitoneal tissue adhesions were observed (red arrow); scale bar: 1 cm. (**B**) Cell-dissolving (black frames; the magnification of each frame is listed on the bottom-right) and many inflammatory cells (black circle, C57BL/6) were observed. Scale bar: 100 μm.

Mice were sacrificed six days after intraperitoneal injection of 21.5 mg/kg solasonine, and livers were collected for histological analysis. Intraperitoneal tissue adhesion was observed in mice of all four strains treated with 21.5 mg/kg of solasonine, but not in vehicle-treated mice (Figure 4A). Compared to other strains, BALB/c mice presented more severe intraperitoneal tissue adhesion (Figure 4A, BALB/c). Consistently, cell-dissolving was seen in liver slices of each strain of mice treated with solasonine at doses of 21.5 mg/kg, but not in mice treated with vehicle (Figure 4B). More inflammatory cells were observed in the liver slices of C57BL/6 mice (Figure 4B, C57BL/6). This suggests that a high dose of solasonine could cause damage to contacted tissues and dissolve cells. 

### 2.5. mRNA Levels of Genes of the cyp450 Family in the Livers of Each Strain of Mice Six Days after Intraperitoneal Injection with Solasonine at a Dose of 21.5 mg/kg

As cyp450 family enzymes could play key roles in drug biotransformation and drug pharmacological and toxicological effects, the expression levels of genes of the cyp450 family were evaluated in the livers of male mice from four strains. We compared basal expression levels of the cyp450 family genes in the livers of mice from four strains, and revealed whether solasonine influenced cyp450 family gene expression. Four cyp450 family genes were basally expressed at a high level: cyp1a2, cyp2e1, cyp3a11, and cyp2d11; and three genes (i.e., cyp2d12, cyp2j12, and cyp4f37) were basally expressed at a low level in the livers of male C57BL/6 mice [18]. These genes were selected to be detected at the mRNA level by SYBR real-time PCR assay six days after the mice were treated by intraperitoneal injection with solasonine at a dose of 21.5 mg/kg, or with a vehicle. 

**Figure 5:**
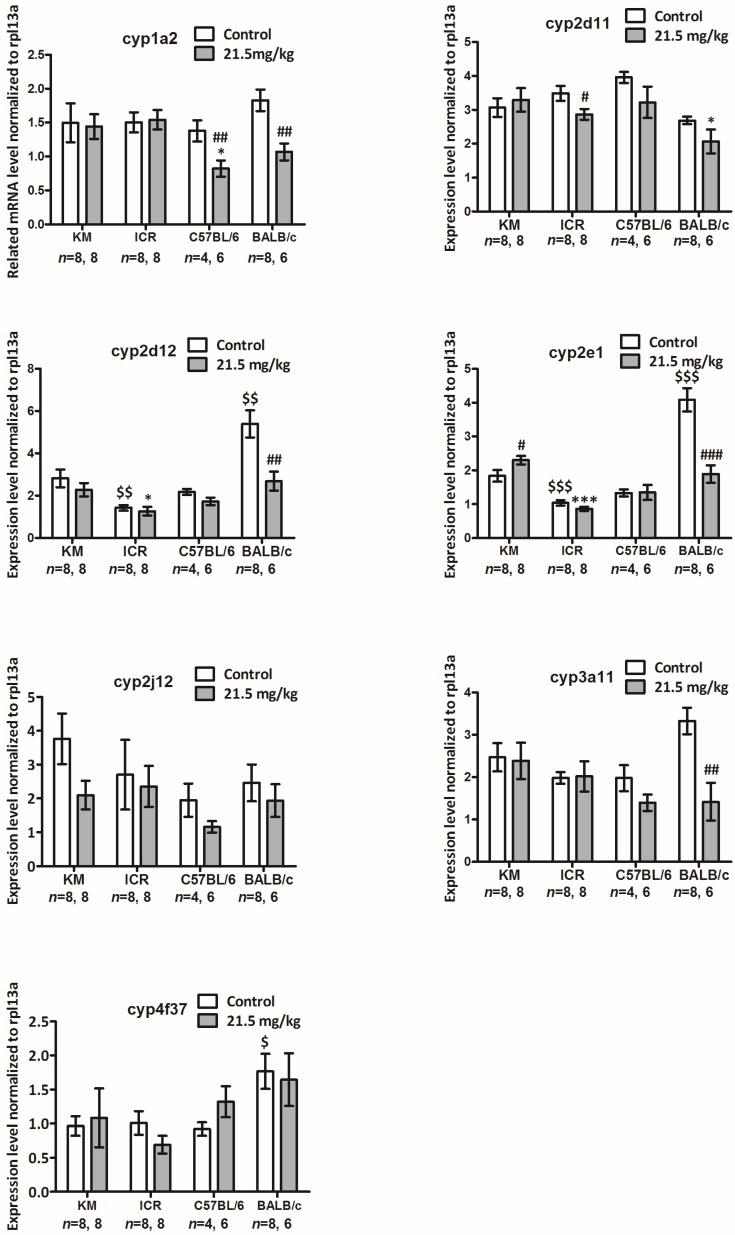
Related mRNA levels of cyp450 family genes in the livers of mice from four strains six days after intraperitoneal injection with solasonine or vehicle (control). Gene expression of each cyp450 family member was normalised to rpl13a mRNA expression. #: compared to vehicle group from the same strain on the same day, #: *p* < 0.05, ##: *p* < 0.01,###: *p* < 0.001; $: compared to vehicle group of KM strain on the same day, $: *p* < 0.05, $$: *p* < 0.01, $$$: *p* < 0.001; *: compared to 21.5 mg/kg group of the KM strain on the same day, *: *p* < 0.05, ***: *p* < 0.001.

For the vehicle group, compared to that of KM mice, cyp2e1 mRNA levels were lower for ICR mice (*p* < 0.001, 0.57 of KM), while they were higher for BALB/c mice (*p* < 0.001, 2.22 of KM, Figure 5, cyp2e1); cyp2d12 mRNA levels were lower for ICR mice (*p* < 0.01, 0.50 of KM), while they were higher for BALB/c mice (*p* < 0.01, 1.91 of KM, Figure 5, cyp2d12); and cyp4f37 mRNA level was higher for BALB/c mice (*p* < 0.05, 1.83 of KM) (Figure 5, cyp4f37).

Compared to the gene expression in livers of mice from the same strain treated with vehicle, in the livers of mice treated with solasonine, cyp1a2 mRNA levels decreased for C57BL/6 (*p* < 0.01, 0.50 of vehicle) and BALB/c strains (*p* < 0.01, 0.58 of vehicle) (Figure 5, cyp1a2); cyp3a11 (*p* < 0.01, 0.43 of vehicle) and cyp2d12 (*p* < 0.01, 0.50 of vehicle) mRNA levels both decreased for BALB/c mice (Figure 5, cyp3a11, cyp2d12); cyp2e1 mRNA levels were higher for KM (*p* < 0.05, 1.25 of vehicle) and lower for BALB/c mice (*p* < 0.001,0.46 of vehicle) (Figure 5, cyp2e1); cyp2d11 mRNA level was lower for the ICR strain (*p* < 0.05,0.82 of vehicle) (Figure 5, cyp2d11). 

Together, these results suggest that, in the livers of male mice, although the basal mRNA levels of most tested genes were no different between these four strains, more genes (4/7) were downregulated at mRNA level by solasonine in the BALB/c strain than those in KM (0/7) and ICR strains (1/7).

### 2.6. Protein Levels of the cyp450 Family Genes in the Livers of Mice from Four Strains Six Days after Intraperitoneal Injection with Solasonine at a Dose of 21.5 mg/kg

To compare the basal expression level of cyp450 family enzymes in the livers of male mice from four strains (i.e., KM, ICR, C57BL/6, and BALB/c), and to reveal whether solasonine influences cyp450 family gene expression at the protein level, five cyp450 family enzymes that are usually targeted in drug research—CYP1A2, CYP3A11, CYP2E1, CYP2D11, and CYP2C19—were detected by ELISA in the livers of male mice treated by intraperitoneal injection with solasonine at a dose of 21.5 mg/kg or with a vehicle.

**Figure 6:**
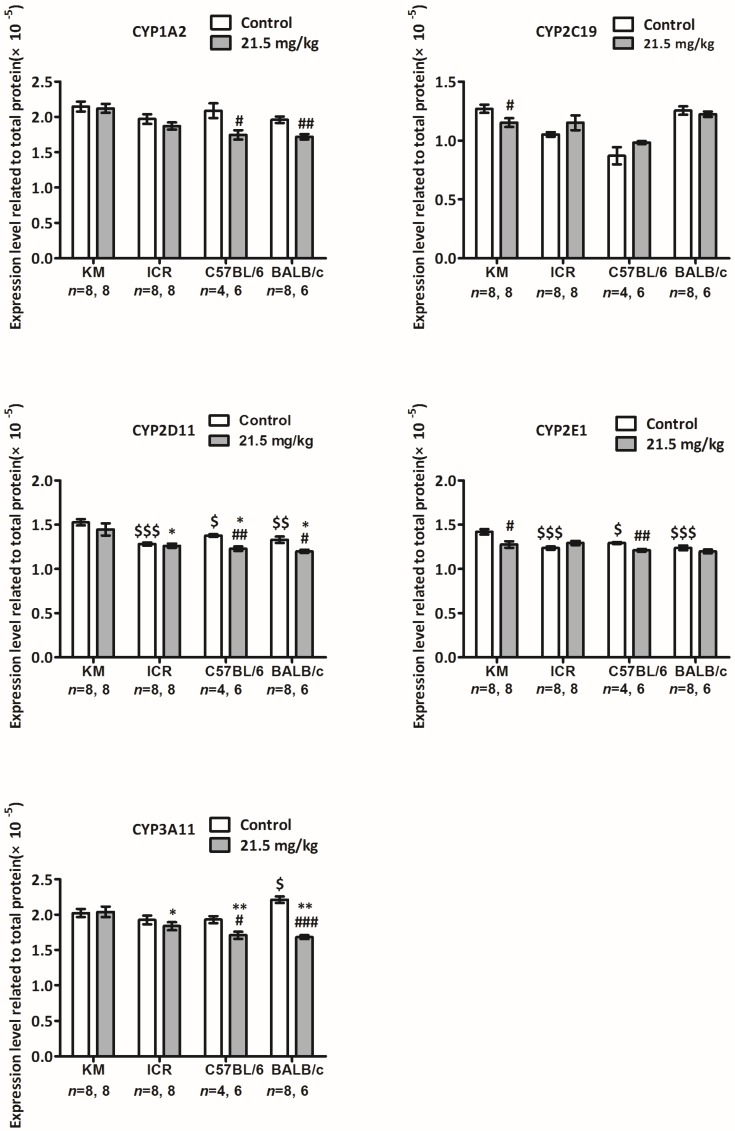
Protein levels of cyp450 family genes in the livers of male mice from four strains 6 days after intraperitoneal injection with solasonine or vehicle (control). Total protein of each sample was diluted in 1× PBS to a final concentration of 2000 ng/mL, and each enzyme was quantified basally on a standard curve established using an ELISA kit. Expression data were normalised to total protein. #: compared to vehicle group from the same strain on the same day, #: *p* < 0.05, ##: *p* < 0.01, ###: *p* < 0.001; $: compared to vehicle group of KM strain on the same day, $: *p* < 0.05, $$: *p* < 0.01, $$$: *p* < 0.001; *: compared to 21.5 mg/kg group of the KM strain on the same day, *: *p* < 0.05, **: *p* < 0.01.

For the vehicle group, compared to KM mice, CYP3A11 levels were higher for BALB/c mice (*p* < 0.05, 1.09 of KM) (Figure 6, CYP3A11); CYP2E1 levels were lower for ICR (*p* < 0.001, 0.87 of KM), C57BL/6 (*p* < 0.05, 0.91 of KM), and BALB/c mice (*p* < 0.001, 0.87 of KM, Figure 6, CYP2E1); CYP2D11 levels were also lower for ICR (*p* < 0.001, 0.84 of KM), C57BL/6 (*p* < 0.05, 0.90 of KM), and BALB/c mouse strains (*p* < 0.01, 0.87 of KM (Figure 6, CYP2D11).

Compared to mice from the same strain treated with vehicle, CYP1A2 levels in the livers of mice treated with solasonine were lower for C57BL/6 (*p* < 0.05, 0.84 of vehicle) and BALB/c strains (*p* < 0.01, 0.88 of vehicle) (Figure 6, CYP1A2); CYP3A11 levels were lower in C57BL/6 (*p* < 0.05, 0.89 of vehicle) and BALB/c mice (*p* < 0.001, 0.76 of vehicle) (Figure 6, CYP3A11); CYP2E1 levels were lower for KM (*p* < 0.05, 0.90 of vehicle) and C57BL/6 mice (*p* < 0.01, 0.94 of vehicle) (Figure 6, CYP2E1); CYP2D11 levels were lower for C57BL/6 (*p* < 0.01, 0.89 of vehicle) and BALB/c (*p* < 0.05, 0.90 of vehicle) (Figure 6, CYP2D11); and CYP2C19 levels were lower in KM mice (*p* < 0.05, 0.91 of vehicle) (Figure 6, CYP2C19).

Together, these data suggest that, in the livers of male mice, although the basal levels of most tested proteins in C57BL/6 and BALB/c strains were not lower than those in KM and ICR strains, more CYP450 proteins were downregulated by solasonine in both C57BL/6 (4/5) and BALB/c (3/5) strains than those in KM (2/5) and ICR (0/5) strains.

## 3. Discussion

The concepts of holism and dialectics are the essence of traditional Chinese medicine (TCM) theory. This theory attaches importance to individual differences in treatment. Moreover, precision treatment and individual treatment have become increasingly common in modern medicine. From the genetic viewpoint, the essences of both individual difference and precision therapy are based on differences in genetic backgrounds. Differences between human races can be viewed as similar to those differences between mouse stains. Therefore, it is reasonable that mouse strains should be utilised as models for research of how genetic backgrounds influence in vivo pharmacological and toxicological functions of drugs and their effects of regulating expression levels of cyp450 family genes, one of the most important gene families for drug metabolism and functions in vivo. In this study, four strains of mice intraperitoneally administered with solasonine showed different toxicological sensitivity to solasonine. The strains, in order of decreasing LD50 value, rescuing speed of body weight, and increasing severity of pathological symptoms, were KM, ICR, C57BL/6, and BALB/c. Interestingly, 6 days after treatment with solasonine at a dose of 21.5 mg/kg, at the mRNA level, four (cyp1a2, cyp2e1, cyp3a11, and cyp2d12) out of seven cyp450 family genes had decreased expression in BALB/c mice, while only one was downregulated in ICR (cyp2d11) and C57BL/6 (cyp1a2) mice. Additionally, no genes were decreased in expression in KM mice (seen in Figure 5). Moreover, at the protein level, three or four (CYP1A2, CYP3A11, CYP2D11 and/or CYP2E1) out of five cyp450 enzymes dropped in BALB/c and C57BL6 mice, and two (CYP2E1 and CYP2C19) out of five enzymes were downregulated in KM mice, while no enzyme was decreased in ICR mice (seen in Figure 6). These results suggest that solasonine has hepatic toxicity and downregulates cyp450 genes expression at transcriptional and/or post-transcriptional levels. Meanwhile, genetic background is an important factor that can affect in vivo toxicity and, thus, should not be ignored. In short, for the in vivo toxicity of solasonine, BALB/c is the most sensitive strain to solasonine, while KM is the least sensitive. Additionally, the C57BL/6 strain was more sensitive to solasonine than the ICR strain. Consistently, more cyp450 family members showed decreased expression in the C57BL/6 and BALB/c strains compared to the KM and ICR strains, suggesting that the downregulation of cyp450 genes expression may be a clue to help understand the mechanism of strain sensitivity to solasonine. As we know, both KM and ICR are outbred strains, while C57BL/6 and BALB/c are inbred strains. This suggests that hybrid superiority may endow individuals with a stronger resistance to drug toxicity, and that the mechanism of this resistance may be related to less cyp450 gene activity in the liver downregulated by drugs. 

In fact, many documents have reported that insufficient cyp450 enzymes can lower the capacity to metabolise drugs. Cyp1a2 was downregulated in the livers of controlled insulin-dependent diabetic mice with more than 10-fold reduction. The livers of controlled insulin-dependent mice showed significantly higher levels of mRNA than uncontrolled insulin-dependent mice, but still lower than the non-diabetic mice; which can at least partly explain the poor drug and fatty acid metabolism in diabetic patients compared to healthy groups [20]. On the other hand, vitamin C can alleviate damage to the liver of weaning piglets by modulating the nuclear receptor signalling pathway and partly elevating levels of phase I metabolic enzyme genes (i.e., cyp1a1, cyp1a2, cyp1a6) [35]. Some minimal basal expression of cypla2 is essential for hexachlorobenzene (HCB)-mediated enhancement of uroporphyria [36]. Malaria can downregulate hepatic levels of cyp1a2, cyp2e1, and cyp3a11 mRNAs, causing prolongation of midazolam sleeping time and a slower clearance of chlorzoxazone [37]. In our study, the expression levels of more cyp450 genes were impaired by solasonine in C57BL/6 and BALB/c mice, than those in KM and ICR mice. Simultaneously, the potency in protecting against the toxicity of solasonine was reduced. This also suggests that solasonine may change the pharmacological and toxicological effects of solasonine itself, and other drugs, by regulating cyp450 genes’ expression.

Dopaminergic systems regulate the release of several hormones, including growth hormone (GH), thyroid hormones, insulin, glucocorticoids, and prolactin (PRL), which play significant roles in the regulation of various CYP450 enzymes. Inhibition of dopamine D2-receptors with sulpiride (SULP) significantly repressed the constitutive and benzo[a]pyrene (B[a]P)-induced cyp1a1, cyp1a2, and cyp1b expression in the rat liver, which appears to be mediated by activation of the insulin/PI3K/AKT pathway [38]. Injection of 5,7-DHT decreased serotonin concentration in the brain, followed by a significant rise in the levels of growth hormone, corticosterone, and testosterone, and a drop in triiodothyronine concentration in the serum. Simultaneously, the activity and protein level of liver cyp1a, cyp3a1, and cyp2c11 rose, and the mRNA levels of cyp1a1, cyp1a2, cyp2c11, and cyp3a1 were also elevated. This suggests hypothalamus–pituitary–secretory organ axes play key roles in CYP450 enzymes’ expression [39]. Moreover, GH and insulin-like growth factor-1 (IGF-1) levels after exercise depended on the work done, and the relative levels of CYP1A2 expression correlated with the time and the amount of work done by athletes [40]. However, tetrahydroxystilbene glucoside (TSG), the main active component of *Polygonum multiflorum*, has inhibitory effects on mouse liver CYP1A2, CYP2E1, and CYP3A11 protein expression through the suppression of aryl hydrocarbon receptor (AhR), pregnenolone X receptor (PXR), and proliferate-activated receptor α (PPARα) activation [19]. In addition, liver insufficiency also impairs CYP450 enzymes’ expression. In a rat model of liver insufficiency with a dysfunctional serotonergic system, cyp1a2 gene expression was simultaneously downregulated, with a concomitant decrease in CYP1A2 protein and activity, a significant reduction in thyroid hormone β (TRβ) receptor levels, together with a simultaneous increase of thyroid hormone α (TRα) receptor gene and protein level (mainly TRα2 isoform) after serotonergic system dysfunction, suggesting that the serotoninergic system is involved in the regulation of CYP1A isoforms without influence from thyroid hormones during liver insufficiency [41]. Downregulation of AhR during the progression of liver fibrosis is associated with decreased expression levels of phase I and II enzymes and drug transporters during inflammation-related signal transduction between AhR and other nuclear receptors [42]. Furthermore, *trans*-factors and *cis*-elements, and others, regulate hepatic CYP450 enzymes’ expression. CpG sites [43], DNA methylation [44], microtubule-interfering agents (through restricting aryl hydrocarbon receptor-mediated c-jun-N-terminal kinase and glucocorticoid receptor) [45], and clock gene mPer2 [46], all influence cyp1a2 expression post-transcriptionally or pre-translationally. Meanwhile, β-catenin is necessary for both cyp1a2 and cyp2e1 expression [47]. At the post-translational level, SUMOylation, enhancing cyp2e1 protein stability and activity, may have important implications in alcoholic liver disease (ALD) [48]. Cytoplasmic heat shock protein 90 (HSP90) and membrane-bound CYP2E1 may directly interact with each other as partner proteins, leading to the dissociation of the CYP2E1 from the membrane, consequently making it possible to transfer microsomal CYP2E1 in complex with HSP90 to the proteasome for proteolysis [49]. As summarised in Reference [21], there are three major neuroendocrinological pathways involved in the stress-mediated regulation of various CYP450 genes in the liver: the hypothalamic–pituitary–GH, hypothalamic–pituitary–adrenal, and hypothalamic–pituitary–thyroid hormone axes. In our study, mice could be stimulated by high levels of solasonine to have post-excitatory depression in 1.5 min, and then showed different toxic symptoms. This phenomenon may be explained by the fact that the depression of the neuroendocrine axis constrains the expression of CYP450 enzymes. KM or ICR mice appear more tolerant to such stimulation than C57BL/6 or BALB/c mice, and thus recover faster in body weight, and so on. These should be further revealed in future investigations.

## 4. Materials and Methods 

### 4.1. Animals

Male SPF (specific pathogen-free) mice of each strain—KM, ICR, C57BL/c, or BALB/c—were purchased from the Hunan Slack Landscape Laboratory Animal Co., Ltd. (Changsha, China). Mice were bred and maintained under specific pathogen-free conditions, and experiments were conducted in accordance with the Institutional Animal Care and Use Committee at the animal facility of the Laboratory Animal Research Center for Science and Technology of Jiangxi University of Traditional Chinese Medicine, China. All experiments were approved by the Animal Care and Use Committee of Jiangxi University of Traditional Chinese Medicine (identification code: JZLLSC2017-032; date of approval: 11 August 2017).

#### 4.1.1. Horn’s Assay (n = 5, 2.15×)

For each strain, 20 male mice, aged 6 weeks, with an average weight (shown as average ± SD) on the day mice were treated of 24.44 ± 0.89 g for KM, 28.88 ± 2.05 g for ICR, 18.43 ± 0.70 g for C57BL/6, and 23.23 ± 0.96 g for BALB/c mice were equally divided into four groups, and then each group was treated by intraperitoneal injection with solasonine (Desite, Chengdu, China, LOT:DST170828-005,CAS:19121-58-5, extracted from the fruits of *Solanum nigrum* Linn., HPLC purity ≥99%, first dissolved in DMSO, then diluted in 1× PBS with appropriate amount of HCl, final pH 6.0–6.5) at doses of 100, 46.4, 21.5, or 10 mg/kg. After treatment, mice were weighed every 2 days, and the numbers of deceased mice were recorded every day, and added up at the end of the week for this study. Then, the LD50 value of the drug on each strain was given according to the statistical analysis table.

#### 4.1.2. In Vivo Toxicity Experiment 

For each strain, male mice, aged 6 weeks, with an average weight (shown as average ± SD) on the day mice were treated of 22.24 ± 1.61 g for KM (*n* = 16), 23.8 ± 1.13 g for ICR (*n* = 16), 21.44 ± 0.65 g for C57BL/c (*n* = 10) and 19.08 ± 0.71 g for BALB/c (*n* = 17) mice were randomly divided into two groups, and then each group was treated by intraperitoneal injection with the above solasonine at a dose of 21.5 mg/kg or the above vehicle. After treatment, mice were weighed every 2 days. The numbers of deceased mice were recorded every day, and added up at the end of the week for this study. On day 6 after solasonine treatment, all mice were sacrificed, and tissues were collected for studies. Liver, spleen, and body weight were taken.

### 4.2. Haematoxylin–Eosin Staining

Liver was dissected, fixed in cold 4% paraformaldehyde (PFA) overnight at 4 °C, dehydrated by an alcohol gradient (from 50% to 100%), rendered transparent in xylol, and embedded in paraffin. Then, tissue was cut on a microtome to slices of 5 µm in thickness. After deparaffinisation and rehydration, slices were stained with haematoxylin (Solarbio, Beijing, China), dehydrated by an alcohol gradient (from 50% to 95%), stained with eosin (Solarbio, Beijing, China), further dehydrated by 100% alcohol, rendered transparent in xylol, embedded in neutral resin, and finally covered by a cover slip. Samples were imaged by a biomicroscope (Leica, Wetzlar, Germany).

### 4.3. RNA and Real-Time PCR

Livers were homogenised by an electric homogeniser in TRIquick (Solarbio, Beijing, China). Total RNA samples were isolated and reverse-transcribed to cDNA using a TRUEscript 1st Strand cDNA Synthesis kit (Aidlab Biotechnlologies Co., Ltd., Beijing, China) according to the manufacturer’s instructions. Diluted cDNAs were used for real-time PCR with SYBR Green reagents (Mei5 Biotechnology, Co., Ltd., Beijing, China) on a bioanalyser (Roche LightCyler96, IN, USA). Sequences of primers referred to in the document [17] are listed in Table S1. Expression data were normalised to rpl13a mRNA expression. Expression changes were calculated using the ∆∆Ct method.

### 4.4. Protein and ELISA (Enzyme-Linked Immunosorbent Assay)

Livers in 1× PBS were homogenised by an electric homogeniser in ice water. Total protein in each sample was quantified by a total protein detection kit (Aidlab Biotechnlologies Co., Ltd., Beijing, China), and then diluted in 1× PBS to a final concentration of 2000 ng/mL. Optical density (OD) values of enzymes in each sample were detected on a microplate reader (Thermo, VARIOSKAN FLASH, MA, USA) using an ELISA Kit (He Peng Biotechnology Co., Ltd., Shanghai, China) following the manufacturer’s instructions, and then each enzyme was quantified basally, based on a standard curve that was established using an ELISA kit. Expression data were normalised to total protein at a concentration of 2000 ng/mL.

### 4.5. Statistical Analysis

Data were expressed as the mean ± SEM. Statistical analyses were carried out using GraphPad Prism 5 software (Vesion 5.01, San Diego, CA, USA, 2007) and *t*-test analysis was performed between two groups. All *p*-values less than 0.05 were considered to be statistically significant.

## Figures and Tables

**Table 1:** LD50 value of solasonine in four murine strains according to statistical analysis table of Horn’s assay (*n* = 5, 2.15×).

Item	KM				ICR				C57BL/6				BALB/c			
**Dose (mg/kg)**	100	46.4	21.5	10	100	46.4	21.5	10	100	46.4	21.5	10	100	46.4	21.5	10
**Death Rate**	2/5	2/5	0/5	0/5	5/5	3/5	0/5	0/5	4/5	4/5	1/5	0/5	5/5	4/5	1/5	0/5
**LD50 (mg/kg)**	>43				43				34.8				31.6			
**95% Confidence Limit**	N/A				29.5–62.6				19.2–63				20.5–48.8			

## Supplementary Materials

The following are available online at http://www.mdpi.com/2072-6651/10/12/487/s1. Figure S1: Changes in body weight of male mice from four strains over one week, Table S1: Primers for real-time PCR of cyp450 family genes and rpl13a, Movie S1: Behavioural response of mice to 21.5 mg/mL solasonine or vehicle after intraperitoneal administration (seen or downloaded in the site: http://susy.mdpi.com/user/submission/video/87f3d40f66418381426f9f271272b6be).
