# Supplementary material for: In Vivo Toxicity of Solasonine and Its Effects on cyp450 Family Gene Expression in the Livers of Male Mice from Four Strains
Source: Toxins (Basel). 2018 Nov 23;10(12):487. doi: 10.3390/toxins10120487 (PMC6315709; doi:10.3390/toxins10120487)
Supplement: Supplementary file 1 [file toxins-10-00487-s001.pdf]

Supplementary Materials: In Vivo Toxicity of Solasonine and Its Effects on cyp450 Family Gene Expression in the Livers of Male Mice from Four Strains

Youbao Zhong, Shanshan Li, Liling Chen, Zhiyong Liu, Xiaoquan Luo, Peng Xu and Lai Chen

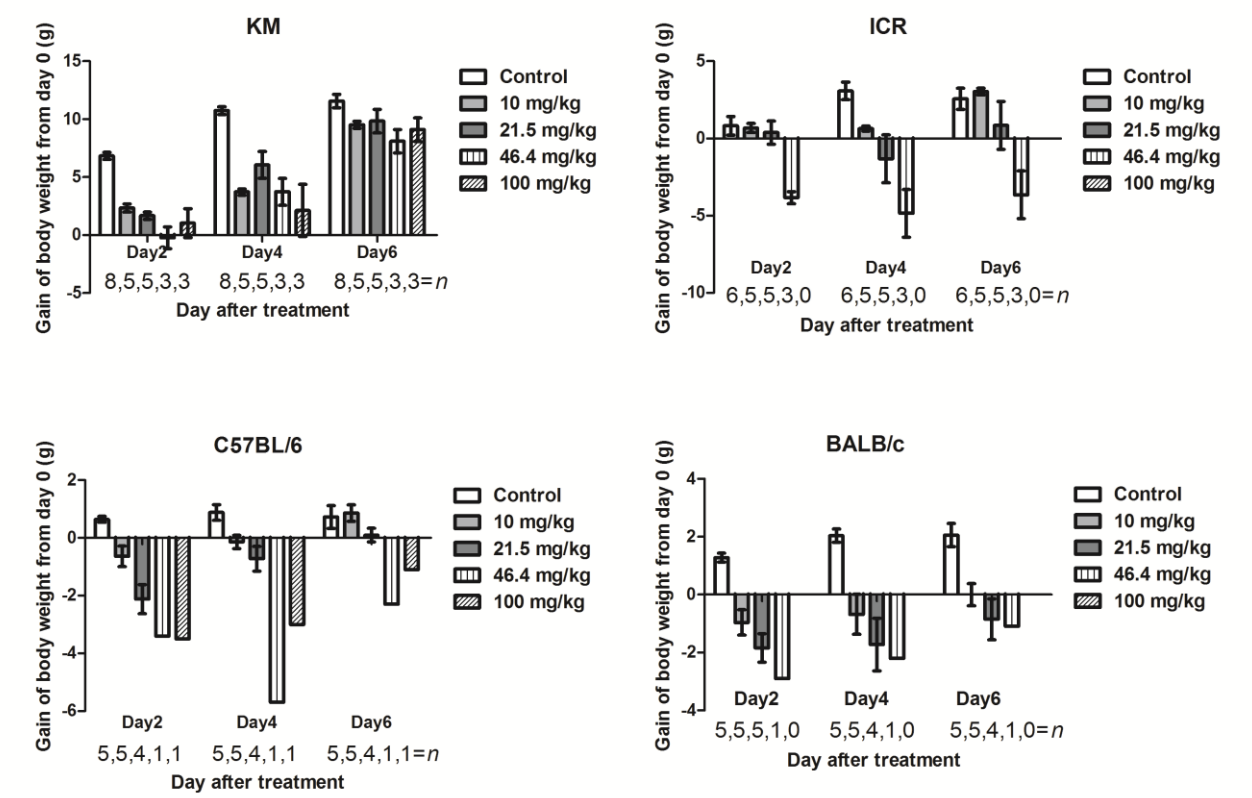

Figure S1. Changes in body weight of male mice from four strains over one week. Mice of each strain—KM, ICR, C57BL/6, or BALB/c—were treated by intraperitoneal injection with solasonine at a dose of 100, 46.4, 21.5, or 10 mg/kg, or with vehicle (control), and then the body weight of each living mouse was weighed and recorded and the average increase in body weight of each mouse from Day 0 were shown.

Table S1. Primers for real-time PCR of cyp450 family genes and rpl13a.

Gene	Direction	Primer Sequence
cyp1a2	F	CCTCTATGCCAACACGTGC
	R	CCTGCTTGCTGATCCACATC
cyp2e1	F	TGGGGAAACAGGGTAATGAG
	R	GCACAGCCAATCAGAAAGGT
cyp3a11	F	AGAAGGCAAAGAAAGGCAAG
	R	CTCTGGGTTGTTGAGGGAAT
cyp2d11	F	TCTCAGTGCCTGATGGACAG
	R	CACAGAGCTGGTAGGGGAAG

cyp2d12	F	ACACTGCTGACCGCCCTCCA
	R	TGCTCTCGCCACTCAGGTCCA
cyp4f37	F	ACTGAAGCAGGCCAGACTTACCG
	R	GGGGGCAACAAATGCAGGGTCA
cyp2j12	F	CTGATAGCACACTGGCTGGA
	R	GGTCCCTGTGAAGAGCTGTC
rpl13a	F	ACAAGAAAAAGCGGATGGTG
	R	TTCTCCTCCAGAGTGGCTGT
